# Supplementary figures and images for: Hidden hematological, biochemical and immune costs of asymptomatic malaria infections in semi-wild chimpanzees
Source: bioRxiv. 2025 Nov 4:2025.11.04.686503. Preprint. [Version 1] doi: 10.1101/2025.11.04.686503 (PMC12637481; doi:10.1101/2025.11.04.686503)

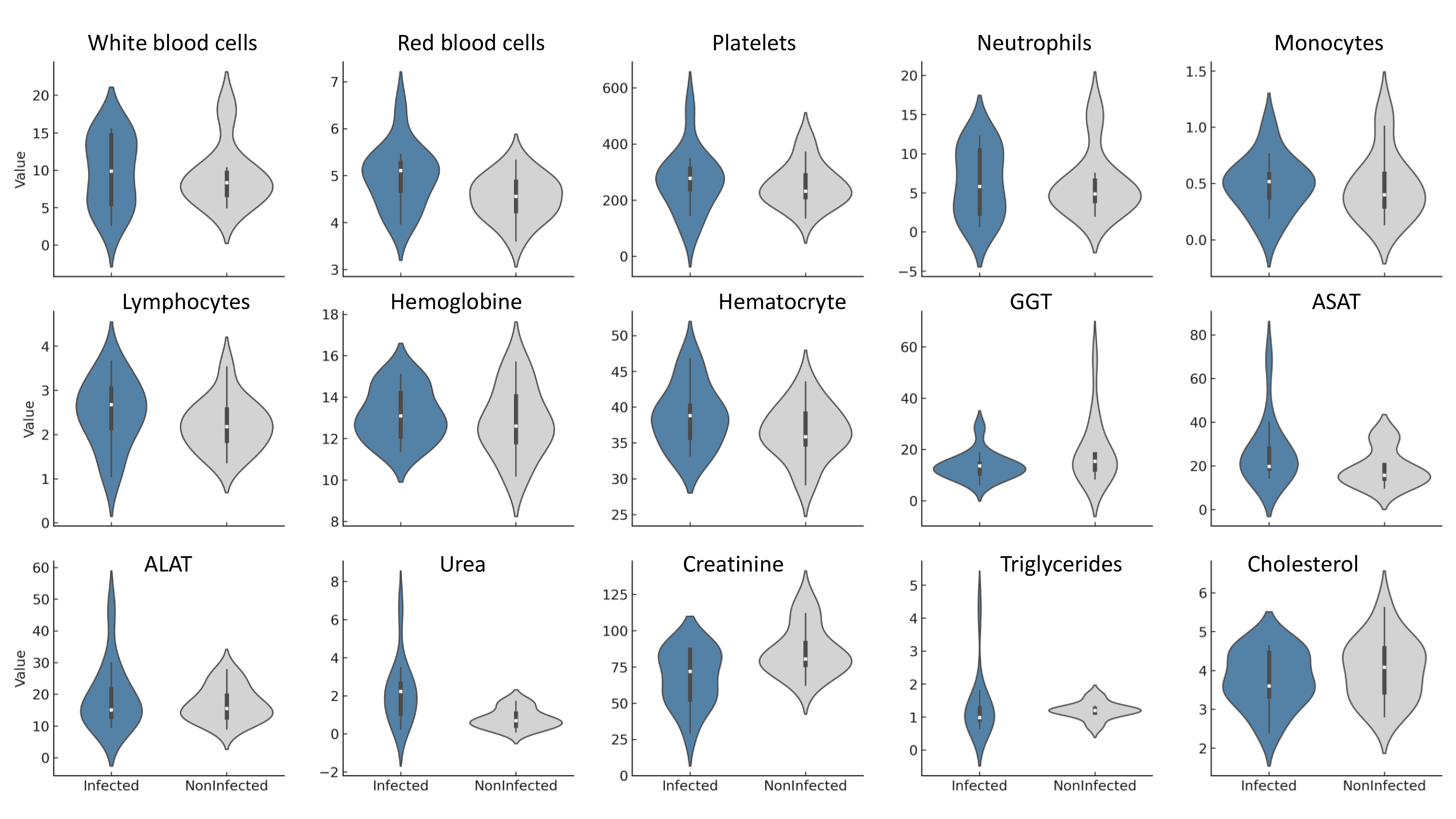

Supplement: Supplement 2 [file media-2.jpg]

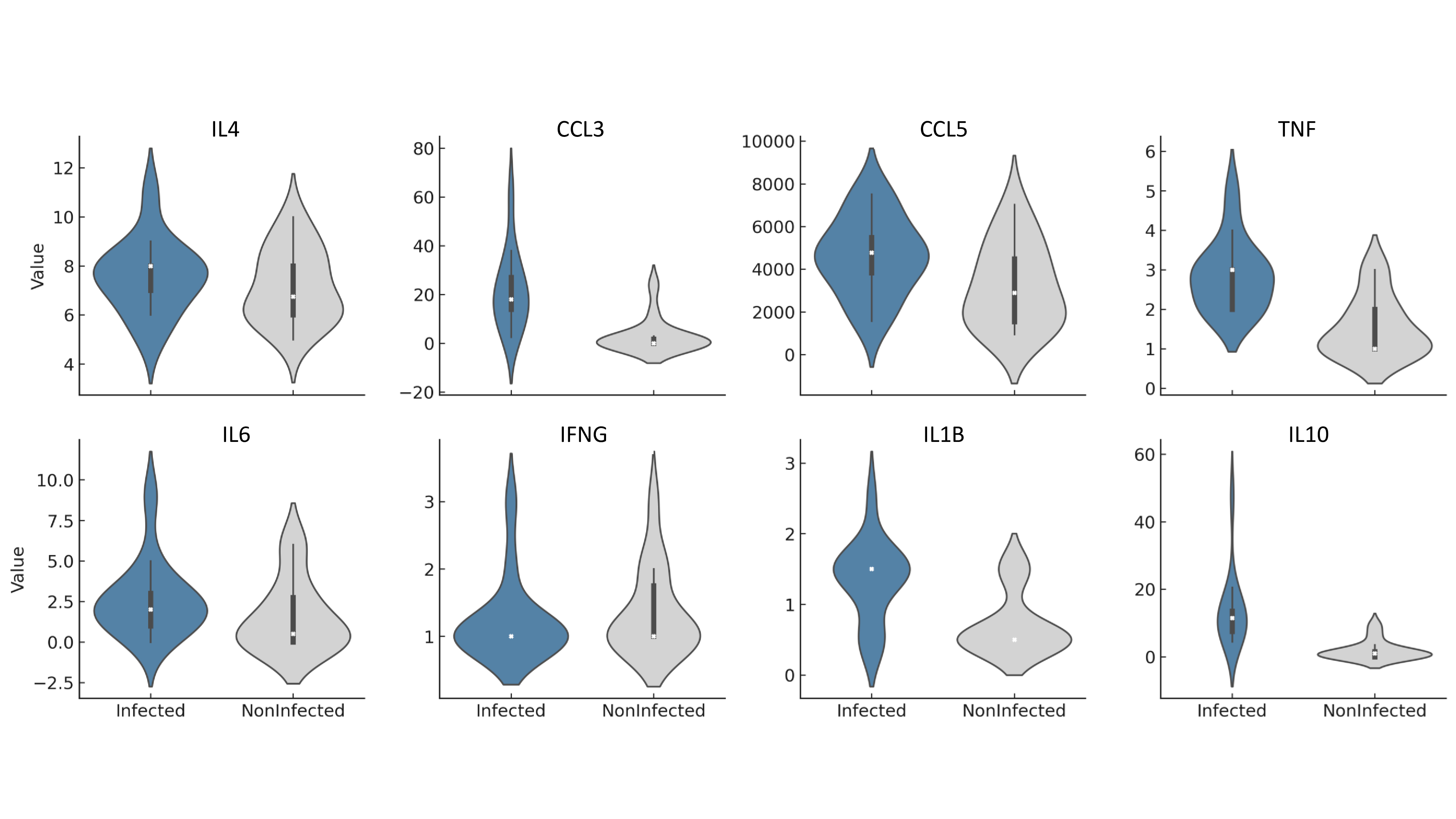

Supplement: Supplement 3 [file media-3.jpg]

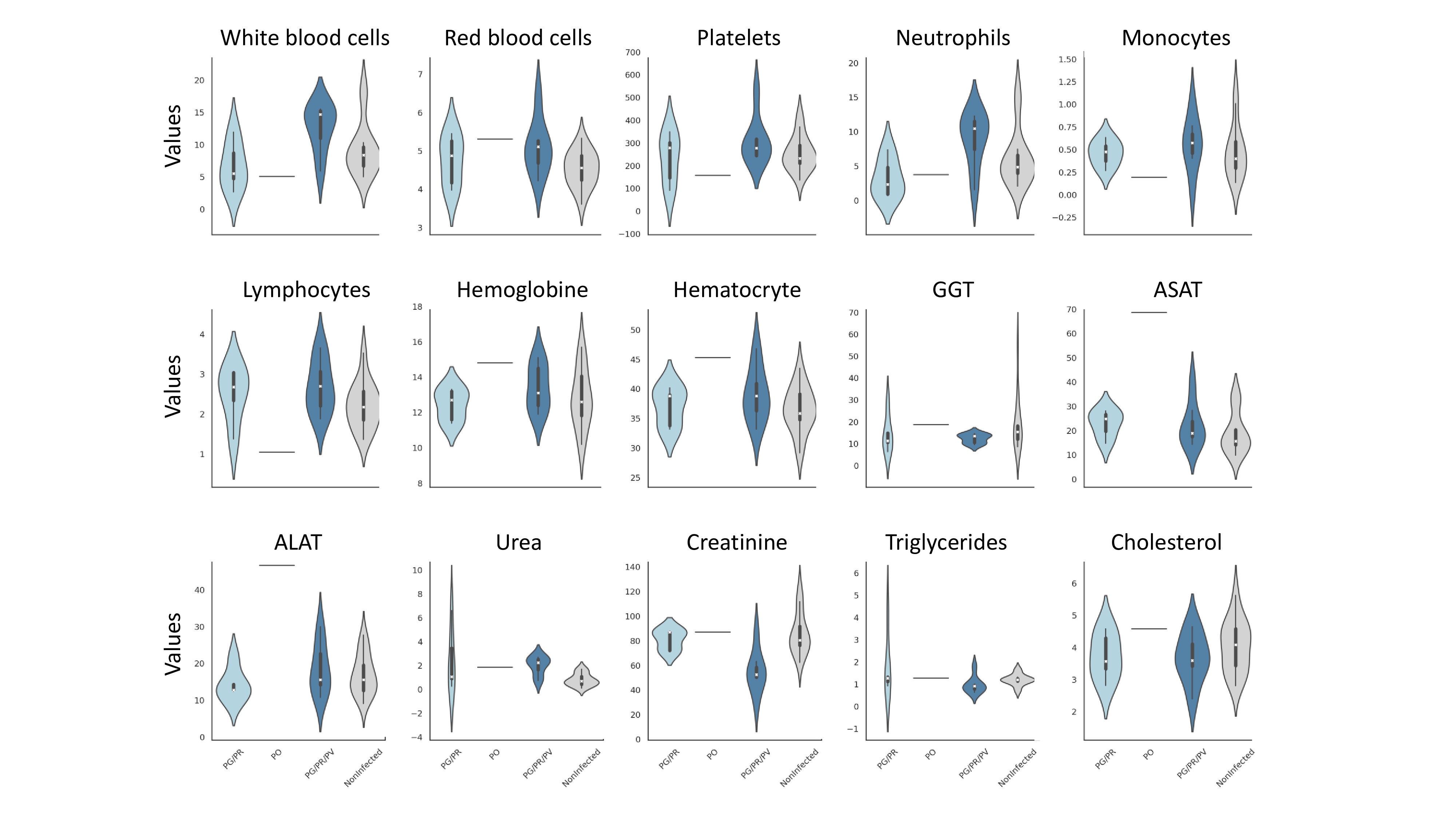

Supplement: Supplement 4 [file media-4.jpg]

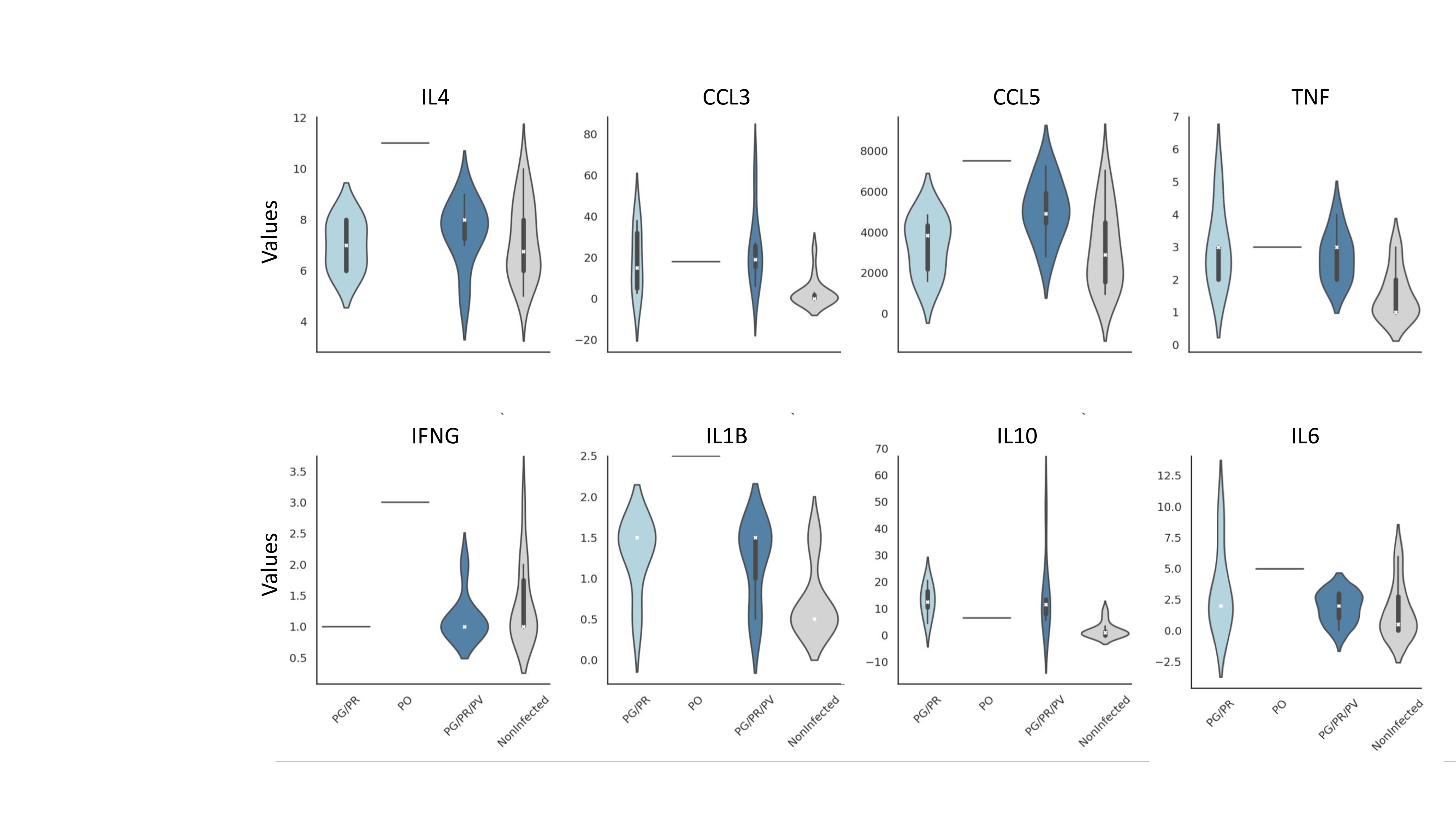

Supplement: Supplement 5 [file media-5.jpg]
